# Evaluation of Internet-Based Dengue Query Data: Google Dengue Trends

**DOI:** 10.1371/journal.pntd.0002713

**Published:** 2014-02-27

**Authors:** Rebecca Tave Gluskin, Michael A. Johansson, Mauricio Santillana, John S. Brownstein

**Affiliations:** 1 Children's Hospital Informatics Program, Children's Hospital Boston, Boston, Massachusetts, United States of America; 2 Dengue Branch, Division of Vector-Borne Diseases, Centers for Disease Control and Prevention, San Juan, Puerto Rico; 3 School of Engineering and Applied Sciences, Harvard University, Cambridge, Massachusetts, United States of America; Emory University, United States of America

## Abstract

Dengue is a common and growing problem worldwide, with an estimated 70–140 million cases per year. Traditional, healthcare-based, government-implemented dengue surveillance is resource intensive and slow. As global Internet use has increased, novel, Internet-based disease monitoring tools have emerged. Google Dengue Trends (GDT) uses near real-time search query data to create an index of dengue incidence that is a linear proxy for traditional surveillance. Studies have shown that GDT correlates highly with dengue incidence in multiple countries on a large spatial scale. This study addresses the heterogeneity of GDT at smaller spatial scales, assessing its accuracy at the state-level in Mexico and identifying factors that are associated with its accuracy. We used Pearson correlation to estimate the association between GDT and traditional dengue surveillance data for Mexico at the national level and for 17 Mexican states. Nationally, GDT captured approximately 83% of the variability in reported cases over the 9 study years. The correlation between GDT and reported cases varied from state to state, capturing anywhere from 1% of the variability in Baja California to 88% in Chiapas, with higher accuracy in states with higher dengue average annual incidence. A model including annual average maximum temperature, precipitation, and their interaction accounted for 81% of the variability in GDT accuracy between states. This climate model was the best indicator of GDT accuracy, suggesting that GDT works best in areas with intense transmission, particularly where local climate is well suited for transmission. Internet accessibility (average ∼36%) did not appear to affect GDT accuracy. While GDT seems to be a less robust indicator of local transmission in areas of low incidence and unfavorable climate, it may indicate cases among travelers in those areas. Identifying the strengths and limitations of novel surveillance is critical for these types of data to be used to make public health decisions and forecasting models.

## Author Summary

Dengue is a common and growing problem worldwide. Delays in traditional surveillance systems limit the ability of public health agencies to identify and respond to dengue outbreaks efficiently. Internet search queries provide near real-time indicators of infectious disease activity and have proven effective for monitoring disease activity in some countries, but have not been assessed on smaller geographic areas. We compared Google Dengue Trends data for 17 states in Mexico to traditional surveillance data from those states. We found that the utility of Google Dengue Trends at the state-level is highly variable and depends on climatic conditions supporting dengue virus transmission. Novel surveillance tools like Google Dengue Trends can provide timely information to public health agencies, but to be useful on a local scale, they must be considered within the local context of dengue transmissibility.

## Introduction

The global incidence of dengue has increased 30-fold between 1960 and 2010 [1], with a recent study estimating that there are now 70–140 million cases per year [2]. Dengue is caused by infection with any of the four dengue virus (DENV) serotypes; the symptoms often include high fever, intense joint and muscle pain, headaches, and skin rash. Some infections result in more serious illness including hemorrhagic symptoms and death [3]. Endemic in many Asian and Latin American countries, dengue has become a leading cause of hospitalization and death among children in these regions [4] and contributes to substantial economic loss for governments and households [5]. Despite the health and economic impacts of dengue, population-level control methods are limited, resource intensive, and largely ineffective to date. Real-time dengue surveillance, therefore, is critical for identifying areas where transmission is ongoing or likely to occur so that interventions can be optimized.

Traditional, healthcare-based, government-implemented dengue surveillance has several shortcomings. Often, it takes weeks to aggregate surveillance data and publish related reports. This lag in part reflects the time needed to collect and aggregate data at different scales, from practitioners up to the Ministry of Health level, but it can also be delayed or interrupted due to lack of resources and bureaucratic or political changes [6], [7]. Meanwhile, as global Internet use has increased, novel disease monitoring tools based on health-related search queries have emerged. Google Dengue Trends (GDT) was developed by aggregating historical logs of anonymous online Google search queries associated with dengue using the methods developed for Google Flu Trends, a tool created to estimate influenza rates [8]. Google queries have shown to be a close proxy for national-level dengue surveillance in multiple countries [9], [10]. And because data are collected and processed in near real-time, these tools produce surveillance data much faster than traditional systems [8], [11], [12]. While GDT has this significant advantage and well-demonstrated large-scale accuracy, it remains unclear how well it works at smaller scales where dengue transmission may be more heterogeneous.

Dengue transmission dynamics are sensitive to the environmental factors that affect the vector mosquitoes [13]. Temperature increases can decrease the length of the gonotrophic cycle [14], increase the feeding frequency [15], increase the rate of mosquito development, and reduce the length of the DENV incubation period within the mosquito [16], [17]. Mosquito survival also increases with temperature, but at a certain point, high temperatures can also lead to high mosquito mortality [14], [18], [19]. Precipitation is also important to the spatial and temporal spread of the mosquito vector [20]–[24]. Lastly, human behavior and habitat modification can contribute to DENV transmission dynamics: the use of screens or air conditioning can reduce human-vector contact [13]; water storage and trash disposal practices are important determinants of larval habitat availability [25]; and a high human population density provides more transmission opportunities [26]. Therefore, information about relevant environmental conditions can contribute to identifying the dengue risk.

Mexico provides a unique setting to assess the value of GDT data; the climate varies widely across the country, dengue is endemic in many areas yet largely absent in others, and approximately 36% of the population has Internet access [27]. Here, we explore the relationship between GDT data and traditional surveillance data for 17 states in Mexico and use climate and socio-demographic data to investigate geographic variation in GDT accuracy.

## Methods

The GDT index was developed as a linear model to predict reported dengue incidence from dengue-related Internet search patterns [9]. Specifically, it incorporates weekly query volume for key terms (normalized to overall search volume) and uses the historical relationship between those terms and reported cases to linearly predict (nowcast) dengue activity. We downloaded weekly GDT data for 2003–2011 for Mexico as a country and for the available years in that time range (2–8 years) for the 17 individual states with available data: Baja California, Chiapas, Colima, Distrito Federal, Estado de Mexico, Jalisco, Morelos, Nayarit, Nuevo LeÓn, Oaxaca, Quintana Roo, Sinaloa, Sonora, Tabasco, Tamaulipas, Veracruz and Yucatan [28] [9]. To create a monthly GDT variable, we averaged GDT across all weeks beginning in each month.

Traditional monthly dengue surveillance data for the same time period - 2003–2011 - were obtained from the Mexican Secretariat of Health (http://www.epidemiologia.salud.gob.mx/anuario/html/anuarios.html) [29], Long-term (1941–2005) mean annual precipitation (millimeters per year) and mean, minimum, and maximum temperature (°C) data were obtained for each state from the Mexican Secretariat of the Environment and Natural Resources (SEMARNAT) (smn.conagua.gob.mx). State-level socio-demographic data were obtained from the Mexican National Institute of Statistics and Geography (INEGI) (www.inegi.org.mx/). The socio-demographic data included the most recent data available for the following variables: the population size and density per kilometer (2010), the percentage of the population under the age of 15 (2010), the number of doctors per 100,000 residents (2008), the percentage of the population with access to drinking water (2006), the percentage of the population with municipal sewage (2008), the percentage of the population with Internet access (2008), and the average household income in pesos (2010). The data for precipitation, population size, population density, and average yearly dengue cases were log transformed to reduce skewing.

To quantify the accuracy of GDT relative to reported dengue cases, we used Pearson correlation to assess linear correlation because GDT was designed as a linear predictor of dengue incidence. We estimated the association between GDT and the traditional surveillance data at the national level and for each state, and calculated coefficients of determination (R^2^) to assess the proportion of dengue incidence variance captured by the GDT data. We then logit-transformed R^2^ and used Gaussian regression to assess the association between each climate and socio-demographic variable and the variability in state-level correlations between GDT and traditional surveillance data. The Akaike's Information Criterion (AIC) was applied to compare the fit for each of the different models. All calculations were performed in R version 2.14 (http://www.r-project.org/).

## Results

**Figure 1:**
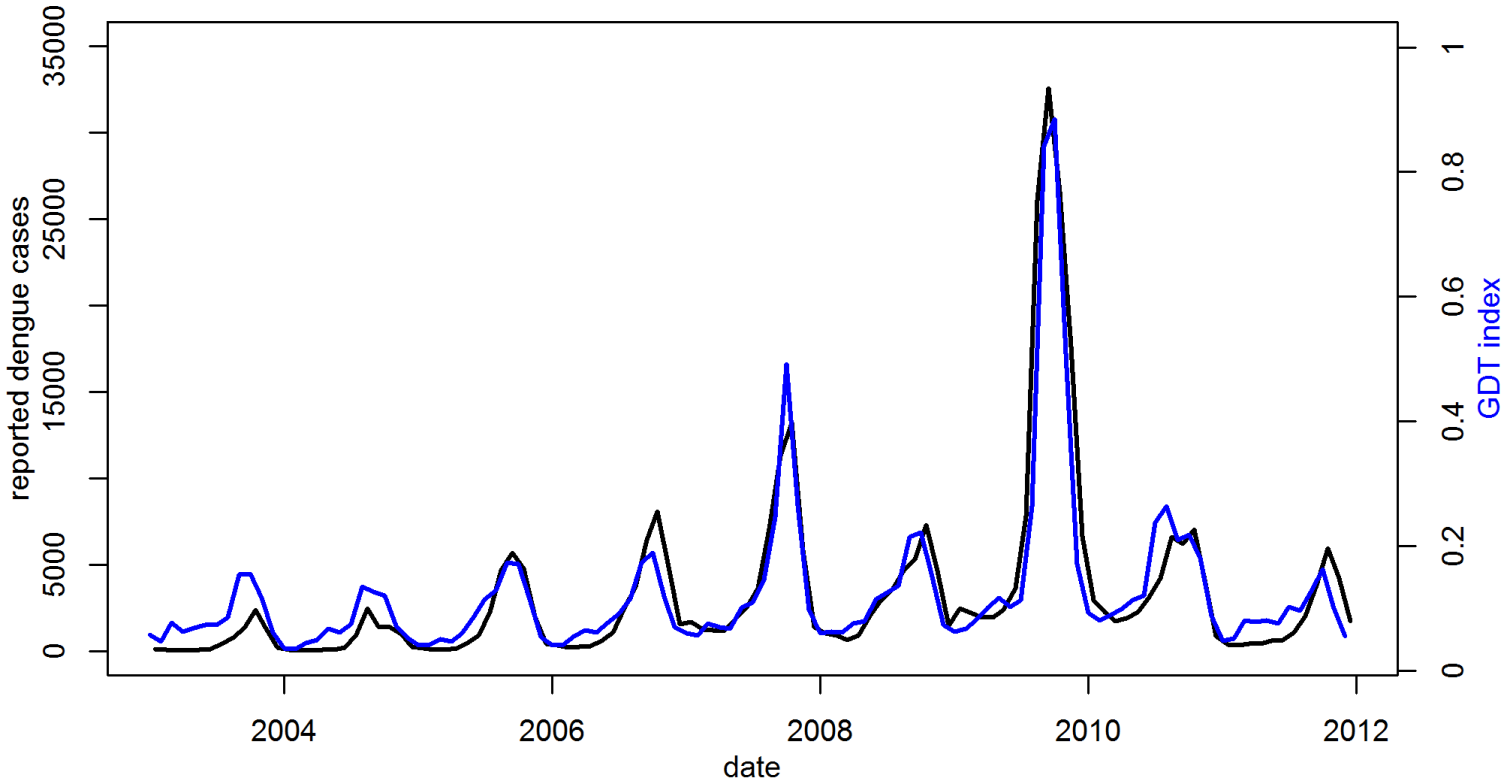
Time Series of monthly reported cases and Google Dengue Trends, Mexico. 2003–2011. The number of cases reported by the Secretariat of Health is shown on the left axis (black) and the GDT index on the right (blue). The correlation coefficient between reported dengue cases and GDT was 0.91 over the 9 years, indicating that GDT captured approximately 83% of the variability in the national surveillance data.

A total of 352,093 dengue cases were reported in all of Mexico from 2003–2011. Figure 1 shows the national-level monthly GDT index compared to the monthly reported cases. These data show a pattern of seasonal outbreaks, generally peaking between August and November, and substantial variation in incidence between seasons. The Pearson's correlation coefficient between GDT and reported dengue cases was 0.91 over the 9 years, indicating that GDT captured approximately 83% of the variability in the national surveillance data.

**Figure 2:**
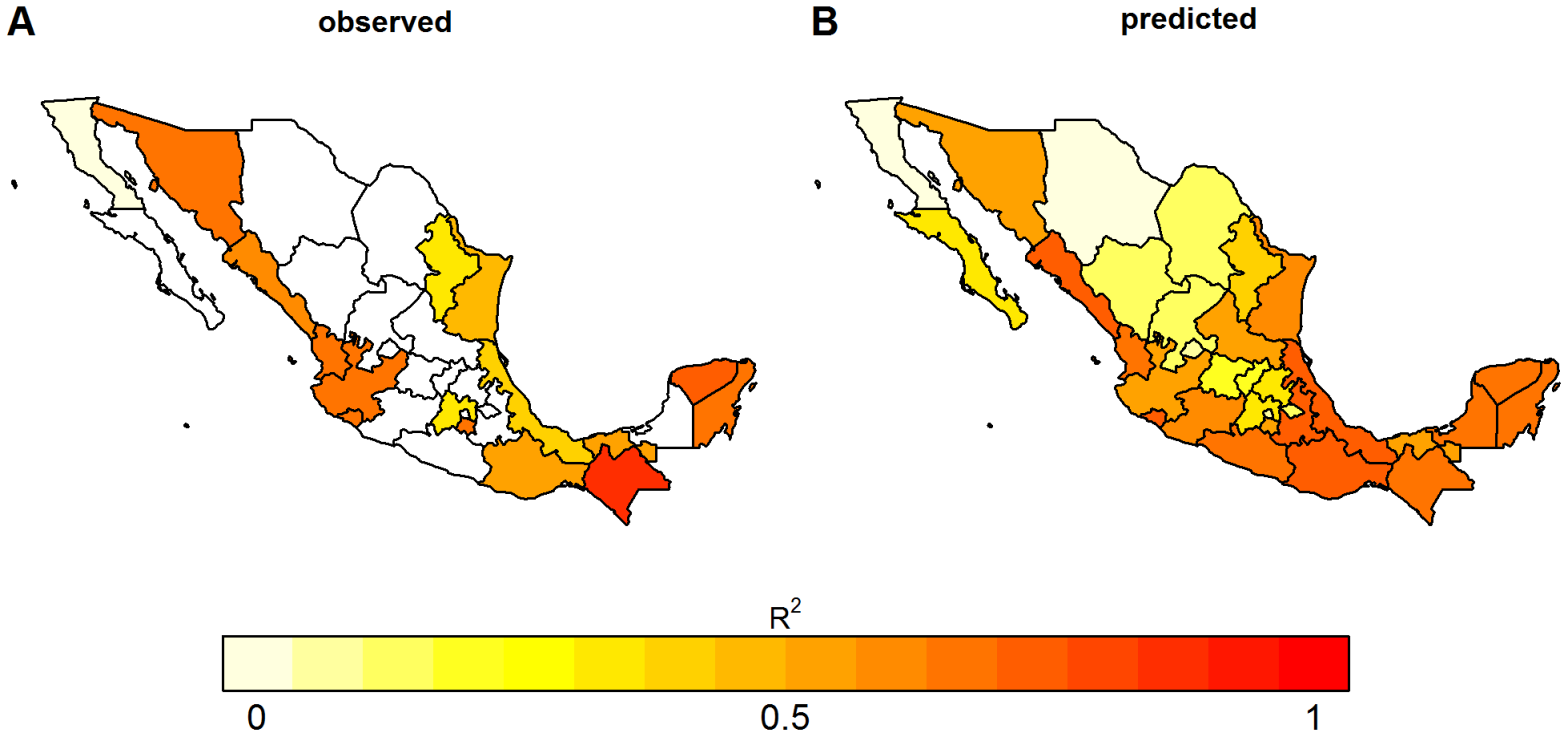
Observed and model-estimated R^2^ for GDT and reported dengue cases. Darker shading indicates a higher coefficient of determination between GDT and traditional surveillance data from observed data (A) and for predictions from the model using maximum temperature, precipitation and the interaction of those two variables (B).

Correlation between monthly GDT and traditional surveillance data, however, varied between states. The coefficient of determination, R^2^, varied from 0.01 in Baja California to 0.88 in Chiapas. Despite the presence of GDT data for the Distrito Federal, the biggest metropolitan area of the country, R^2^ could not be calculated because there were no reported cases during the study period. Figure 2A shows the coefficients of determination for this relationship in each state. In general, there was a stronger correlation in the southern and western coastal states, with the exception of Baja California.

**Figure 3:**
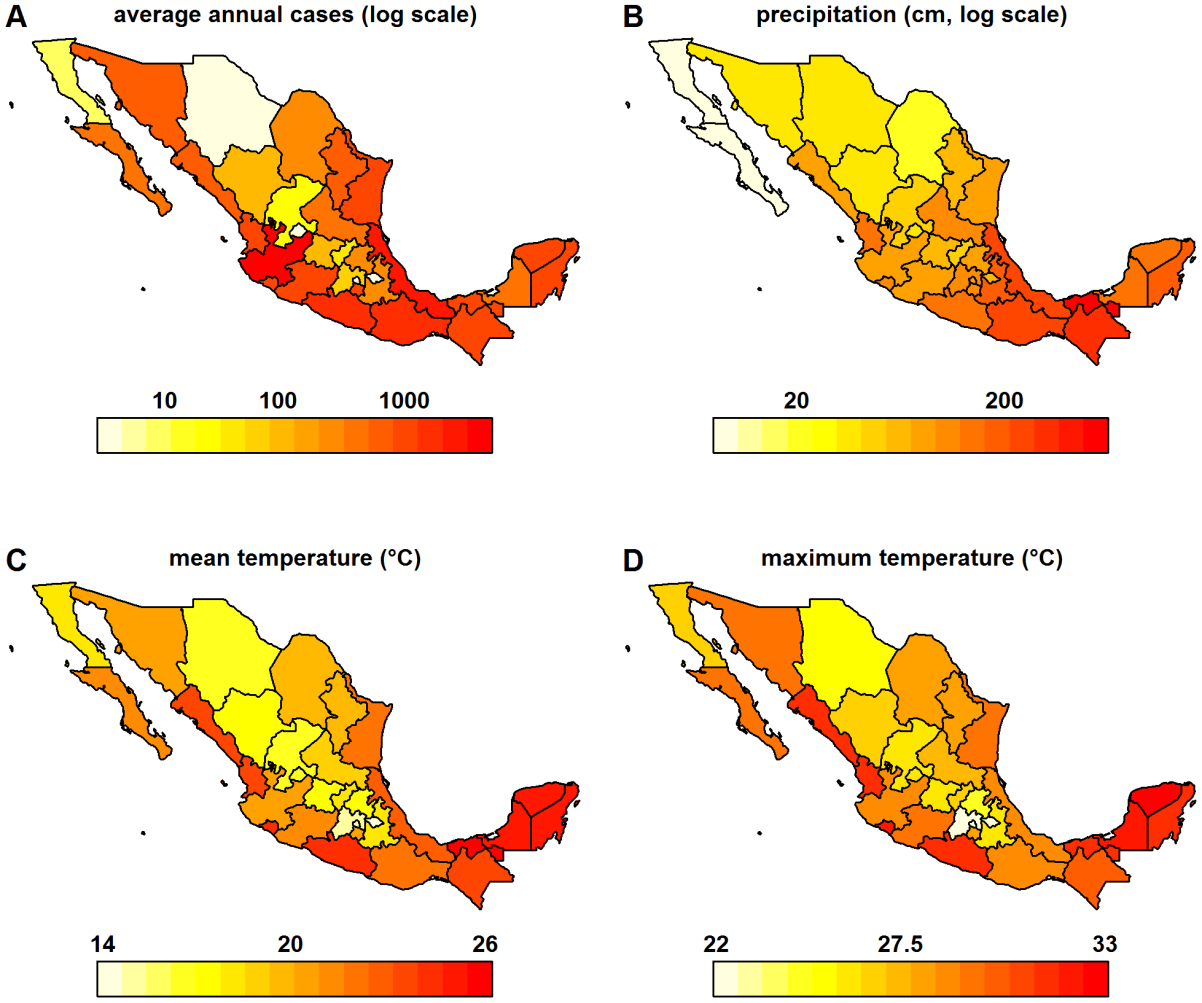
Geographic variation of state-level covariates. The covariates most highly associated with GDT accuracy (Table 1) were average annual dengue cases (A), average annual precipitation (B), mean temperature (C) and maximum temperature (D).

State-level correlation between GDT and case data was strongest in the states with high annual dengue incidence (Table 1, Figure 3A). States with higher average mean temperature, maximum temperature, and precipitation had significantly higher correlation between GDT and dengue case numbers (Figure 3B–D, Table 1). States with lower average household income, a greater proportion of youths in the population, and less internet access tended to have higher correlations, but these associations were not statistically significant (Table 1). We investigated models incorporating combinations of these variables. A model incorporating maximum temperature, logged precipitation, and the interaction of those two variables described 81% of the variance compared to 67% for the model with only dengue incidence and reduced the AIC from 43 to 39 (Table 1, Table 2). Adding socio-demographic factors to this model did not improve the fit.

**Table 1:** Determinants of logit-transformed R^2^ between Google Dengue Trends and government reported dengue cases: single covariate models.

	Coefficient	95% Confidence Interval	R^2^	AIC^b^
**Annual dengue cases** a	**0.61**	**(0.36, 0.86)**	**0.67**	**43**
Minimum temperature	0.18	(−0.02, 0.37)	0.21	57
**Mean temperature**	**0.24**	**(0.01, 0.47)**	**0.26**	**56**
**Maximum temperature**	**0.28**	**(0.02, 0.55)**	**0.27**	**56**
**Precipitation** a	**1.6**	**(0.5, 2.6)**	**0.44**	**52**
Population^a^	−0.4	(−1.3, 0.6)	0.04	60
Population density^a^	−0.05	(−0.9, 0.81)	0	61
Percent youth	0.2	(−0.23, 0.63)	0.07	60
Doctors per 100 k residents	0.01	(−0.01, 0.03)	0.06	60
Potable water	−0.02	(−0.12, 0.07)	0.02	61
Municipal sewage	−0.01	(−0.09, 0.06)	0.01	61
Internet access	−0.06	(−0.15, 0.03)	0.12	59
Household income	−7.2E-05	(−14.5E-05, 0.1E-05)	0.24	57

a Log-transformed.

b Akaike information criterion.

**Table 2:** Determinants of logit-transformed R^2^ between GDT and reported dengue cases: Multiple covariate model.

	Coefficient	95% Confidence Interval	R^2^	AIC^b^
Maximum temperature	4.6	(2.3, 6.8)		
Precipitation^a^	20	(10, 29)		
Interaction	−0.65	(−0.98, −0.32)		
			0.81	39

a Log-transformed.

b Akaike information criterion.

Next, we used this climate-based model to predict the correlation between GDT and case data for all the states, including those where GDT data are not available (Figure 2B). There was general agreement between observed (Figure 2A) and estimated correlation (Figure 2B). Furthermore, the model predicts that for states with higher incidence such as Guerrero, where GDT is not available, GDT may in fact be a good indicator of dengue. However, in states with lower dengue incidence and cooler temperatures, like Chihuahua, GDT may not be an accurate indicator of dengue incidence. Overall, the results show that GDT is a better indicator of real-time incidence in states with high incidence and climate conditions that favor transmission.

## Discussion

At the national level, we found that the official case reports correlated well with GDT. Yet, the correlation between GDT and reported cases varied substantially from state to state, with stronger correlation in states with higher dengue incidence. Climate plays a key role in determining the geographic range and activity of the mosquitoes that transmit DENV. We found that in states with warmer temperatures and greater precipitation, such as Chiapas and Jalisco, GDT was strongly correlated with reported dengue incidence.

The role of climate in DENV transmission, however, is complicated by other biological and socio-demographic factors [20]. Here, however, we did not find that socio-economic factors had a strong influence on the accuracy of GDT. This is particularly important because GDT relies on internet searches, and internet access can vary widely in different settings. We found that Internet access from home was not associated with GDT accuracy, suggesting that even with Internet access in the 30% range, search query data may be robust enough to capture population-level disease dynamics. Internet access will likely only increase in the future, leading to the possibility that greater data flow will improve the accuracy of measures such as GDT. While it is possible that income or internet access do affect GDT accuracy in Mexico, their importance may be overshadowed and confounded by climate, the strongest determinant in our analysis. Our intention was to identify relatively static characteristics that relate to the potential utility of tools like GDT. As such, we used covariate data from the single, most recent year or long-term averages. Future work will build on these findings to determine how temporal variation in relevant covariates may be combined with GDT to improve dengue prediction.

Using the climate-based model, we predicted the utility of GDT for the states where the GDT data are not available. For example, in Guerrero, where GDT is currently not available, our model suggests that it would provide a robust estimate of dengue incidence. Yet, for states where dengue cases are rarer, such as in Chihuahua, the predicted utility of GDT is low. In these areas, where GDT appears to be a poor indicator of local transmission levels, it may nonetheless be a good indicator of some level of health-related activity such as travelers becoming sick in endemic areas, returning home, and searching for dengue information on the Internet. This information would be useful for those interested in estimating local disease burden if not local transmission intensity. Thus, GDT may provide different value in distinct climatic or socio-economic contexts.

Dengue transmission patterns are highly variable and difficult to predict; timely dengue surveillance methods like GDT are needed to keep pace with the spread of the disease. We found that GDT is accurate in areas of high incidence and favorable vector climate conditions. While it appears to be a less robust gauge of local transmission in areas of low incidence and unfavorable climate, it may indicate infections among travelers. As the burden of dengue increases and traditional surveillance efforts struggle to keep pace, novel surveillance tools like GDT can provide timely information to public health officials and contribute to real-time predictive models.
